# Supplementary material for: The Contraindications to Combined Oral Contraceptives among Reproductive-Aged Women in an Obstetrics and Gynaecology Clinic: A Single-Centre Cross-Sectional Study
Source: Int J Environ Res Public Health. 2022 Jan 29;19(3):1567. doi: 10.3390/ijerph19031567 (PMC8835169; doi:10.3390/ijerph19031567)
Supplement: Supplementary file 1 [file ijerph-19-01567-s001.zip › ijerph-1544359-supplementary.pdf]

Supplementary Materials

Supplementary S1: The RECORD statement – checklist of items, extended from the STROBE statement, that should be reported in observational studies using routinely collected health data [16,17].

	Item No.	STROBE items	Location in manuscript where items are reported	RECORD items	Location in manuscript where items are reported
**Title and abstract**					
	1	(a) Indicate the study's design with a commonly used term in the title or the abstract (b) Provide in the abstract an informative and balanced summary of what was done and what was found	1	RECORD 1.1: The type of data used should be specified in the title or abstract. When possible, the name of the databases used should be included.<br><br>RECORD 1.2: If applicable, the geographic region and timeframe within which the study took place should be reported in the title or abstract.	1

				RECORD 1.3: If linkage between databases was conducted for the study, this should be clearly stated in the title or abstract.	
**Introduction**					
Background rationale	2	Explain the scientific background and rationale for the investigation being reported	2		
Objectives	3	State specific objectives, including any prespecified hypotheses	2		
**Methods**					
Study Design	4	Present key elements of study design early in the paper	3		
Setting	5	Describe the setting, locations, and relevant dates, including periods of recruitment, exposure, follow-up, and data collection	3		

Participants	6	*(a) Cohort study* - Give the eligibility criteria, and the sources and methods of selection of participants. Describe methods of follow-up.<br><br>*Case-control study* - Give the eligibility criteria, and the sources and methods of case ascertainment and control selection. Give the rationale for the choice of cases and controls.<br><br>*Cross-sectional study* - Give the eligibility criteria, and the sources and methods of selection of participants.<br><br>*(b) Cohort study* - For matched studies, give matching criteria and number of exposed and unexposed<br><br>*Case-control study* - For matched studies, give matching criteria and the number of controls per case	3	RECORD 6.1: The methods of study population selection (such as codes or algorithms used to identify subjects) should be listed in detail. If this is not possible, an explanation should be provided.<br><br>RECORD 6.2: Any validation studies of the codes or algorithms used to select the population should be referenced. If validation was conducted for this study and not published elsewhere, detailed methods and results should be provided.<br><br>RECORD 6.3: If the study involved linkage of databases, consider use of a flow diagram or other graphical display to demonstrate the data linkage process, including the number of individuals with linked data at each stage.	3

Variables	7	Clearly define all outcomes, exposures, predictors, potential confounders, and effect modifiers. Give diagnostic criteria, if applicable.	3	RECORD 7.1: A complete list of codes and algorithms used to classify exposures, outcomes, confounders, and effect modifiers should be provided. If these cannot be reported, an explanation should be provided.	3
Data sources/ measurement	8	For each variable of interest, give sources of data and details of methods of assessment (measurement).<br><br>Describe comparability of assessment methods if there is more than one group	4		
Bias	9	Describe any efforts to address potential sources of bias	5		
Study size	10	Explain how the study size was arrived at	5		
Quantitative variables	11	Explain how quantitative variables were handled in the analyses. If applicable, describe which groupings were chosen, and why	5		

Statistical methods	12	(a) Describe all statistical methods, including those used to control for confounding<br><br>(b) Describe any methods used to examine subgroups and interactions<br><br>(c) Explain how missing data were addressed<br><br>(d) *Cohort study* - If applicable, explain how loss to follow-up was addressed<br><br>*Case-control study* - If applicable, explain how matching of cases and controls was addressed.<br><br>*Cross-sectional study* - If applicable, describe analytical methods taking account of sampling strategy<br><br>(e) Describe any sensitivity analyses	5		

Data access and cleaning methods		..	7	RECORD 12.1: Authors should describe the extent to which the investigators had access to the database population used to create the study population.<br><br>RECORD 12.2: Authors should provide information on the data cleaning methods used in the study.	
Linkage		..		RECORD 12.3: State whether the study included person-level, institutional-level, or other data linkage across two or more databases. The methods of linkage and methods of linkage quality evaluation should be provided.	-
**Results**					
Participants	13	(a) Report the numbers of individuals at each stage of the study (*e.g.*, numbers potentially eligible, examined for eligibility, confirmed eligible, included in	5	RECORD 13.1: Describe in detail the selection of the persons included in the study (*i.e.,* study population selection) including filtering based on data quality, data availability and linkage. The selection of included persons can	-

		the study, completing follow-up, and analysed)<br><br>(b) Give reasons for non-participation at each stage.<br><br>(c) Consider use of a flow diagram		be described in the text and/or by means of the study flow diagram.	
Descriptive data	14	(a) Give characteristics of study participants (*e.g.*, demographic, clinical, social) and information on exposures and potential confounders<br><br>(b) Indicate the number of participants with missing data for each variable of interest<br><br>(c) *Cohort study* - summarise follow-up time (*e.g.*, average and total amount)	5		
Outcome data	15	*Cohort study* - Report numbers of outcome events or summary measures over time<br><br>*Case-control study* - Report numbers in each exposure	6		

		category, or summary measures of exposure<br><br>*Cross-sectional study* - Report numbers of outcome events or summary measures			
Main results	16	(a) Give unadjusted estimates and, if applicable, confounder-adjusted estimates and their precision (e.g., 95% confidence interval). Make clear which confounders were adjusted for and why they were included.<br><br>(b) Report category boundaries when continuous variables were categorized<br><br>(c) If relevant, consider translating estimates of relative risk into absolute risk for a meaningful time period	5		
Other analyses	17	Report other analyses done—e.g., analyses of subgroups and interactions, and sensitivity analyses	9		

**Discussion**					
Key results	18	Summarise key results with reference to study objectives	11		
Limitations	19	Discuss limitations of the study, taking into account sources of potential bias or imprecision. Discuss both direction and magnitude of any potential bias	12	RECORD 19.1: Discuss the implications of using data that were not created or collected to answer the specific research question(s). Include discussion of misclassification bias, unmeasured confounding, missing data, and changing eligibility over time, as they pertain to the study being reported.	12
Interpretation	20	Give a cautious overall interpretation of results considering objectives, limitations, multiplicity of analyses, results from similar studies, and other relevant evidence	11,12		
Generalisability	21	Discuss the generalisability (external validity) of the study results	12		

Other Information					
Funding	22	Give the source of funding and the role of the funders for the present study and, if applicable, for the original study on which the present article is based	13		
Accessibility of protocol, raw data, and programming code		..		RECORD 22.1: Authors should provide information on how to access any supplemental information such as the study protocol, raw data, or programming code.	13

Supplementary S2: Institutional review board research approval

Kingdom of Saudi Arabia
King Saudi University (034)
p.o. Box 7805 Riyadh 11472
Tel: +966 11 467 00 11
Fax: +96611 467 19 92
http://medicalcity.ksu.edu.sa

المملكة العربية السعودية
جامعة الملك سعود (٠٣٤)
ص.ب ٧٨٠٥ الرياض ١١٤٧٢
هاتف: +٩٦٦١١٤٦٧٠٠١١
فاكس: +٩٦٦١١٤٦٧١٩٩٢

جامعة الملك سعود
King Saud University

المدينة الطبية الجامعية
Institutional Review Board

28.01.2021 (15.06.1442)
Ref. No. 21/0112/IRB

To: **Dr. Ghadah Asaad Assiri**
Assistant Professor
Department of Clinical Pharmacy
King Saud University College of Pharmacy
Email: gassiri@ksu.edu.sa
Principal Investigator

Cc: Mansour Mahmoud, Manal Alshyhsni, Walaa Almatri
Co-investigators

Subject: **Approval of Amendment 2 of Research Project No. E-20-5243**
Study Title: "The Prevalence of Contraindications to Combined Oral Contraceptives Among Reproductive-aged Women in Obstetrics and Gynecology Clinic, Riyadh, Saudi Arabia: A Single-Center Cross-sectional Study"
Type of Review: Expedite

Dear Dr. Ghadah Asaad Assiri,

Reference to your request for approval of the amendment done on the above-mentioned research project which was approved on 17 November 2020 (02 Rabi-II 1442), please be informed that the IRB has no objection toward your request to change the date of data collection from 01 January 2019 – 15 December 2020 to 01 January 2018 – 15 December 2020 to reach the required sample size. The IRB has approved your request.

You may continue with the conduct of this study using the above-mentioned modification. Please be informed that in conducting this study, you as the principal investigator, are required to abide by the rules and regulations of the Government of Saudi Arabia, the KSUMC IRB policies and procedures and the ICH-GCP Guidelines.

The IRB mandates regular submission of study progress report every six months by the primary investigator. Otherwise, project approval will be suspended.

Thank you!

Sincerely yours,

Prof. Abdulrahman AlSultan
Chairman of IRB
Health Sciences Colleges Research on Human Subjects
King Saud University College of Medicine
P. O. B ox 7805 Riyadh 11472 K.S.A.
Email: aalsultan1@ksu.edu.sa

/rubie

Supplementary S3: Data collection sheet

The Estimated proportion of Contraindications to Combined Oral Contraceptives Among Reproductive-aged Women in Obstetrics and Gynecology Clinic, Riyadh, Saudi Arabia: A Single-centre cross-sectional Study

- **This survey aims to**
  - Study the estimated proportion of contraindications among reproductive-aged women who are taking combined oral contraceptives.
  - Assess risk factors associated with the use of combined oral contraceptives.

    - We kindly ask you to answer the survey with the accuracy and objectivity.
    - Filling this survey will be considered as consent to use the information provided for medical and scientific purposes.
    - Your information will be handled with extreme confidentiality.

A. Personal information:

Age		
Height		
Weight		
Educational level:	○ Unschooled<br>○ Elementary school<br>○ Secondary school<br>○ High school<br>○ Diploma<br>○ Bachelor's Degrees<br>○ Postgraduate	
Marital status	○ Single<br>○ Married	
If you are married:<br><br>Number of births	0	1 or more

B. Information about combined oral contraceptives

- Which kind of contraceptives do you take now/or did you take in the past?
  - Drospirenone and Estradiol (**Angeliq**®)
  - Ethinyestradiol and Drospirenone (**Yasmin**®)

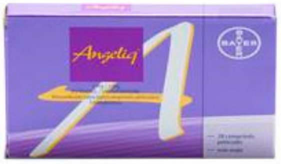

- Are you taking your contraceptive pill regularly?
  - Yes
  - No

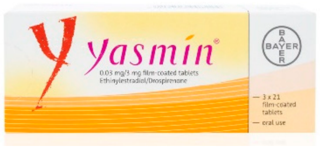

- Are you taking the medication nowadays?
  - Yes
  - No

C. Health status

Please answer the questions

Question	Yes	No	**If the answer is (yes)**		
1. Did you give a birth in the last 21 days?				Less than 21 days	From 21 to 42 days
2. Are you currently breastfeeding?				Less than 6 weeks	From 6 weeks to 6 months
3. Do you smoke cigarettes?			How many cigarettes do you smoke per day?	0-14 Cigarette per day	15 or more
4. Have you been diagnosed with the following			**If the answer is (yes)**		
5. Hypertension.			(If it is available)<br><br>What was your last blood pressure reading?		
6. Deep Vein Thrombosis / Pulmonary Embolism (DVT/PE)			DVT/PE and established on anticoagulant therapy	Yes	No
7. Migraine headaches			Do you suffer from sensory disturbances associated with migraines called aura?<br><br>These disturbances include flashes of light, blind spots, and other changes in vision or twitching in the hand or face)?	Yes	No

8. Breast cancer			**If the answer is (yes)**	Current	Past and no evidence of current disease for five years
9. Diabetes			- Do you have diabetes complications such as Nephropathy, retinopathy, neuropathy?<br>- Diabetes of > 20 years' duration?	Yes	No
10. Known thrombogenic mutations					
11. Current or past gall bladder disease					
12. Systemic Lupus Erythematosus (SLE) with positive (or unknown) antiphospholipid antibodies					
13. Current or history of:<br>- Ischemic heart disease<br>- Stroke<br>- Complicated valvular heart disease					
14. Liver diseases including<br>- Sever cirrhosis<br>- Hepatocellular adenoma<br>- Malignant hepatoma					
15. Acute viral hepatitis					
16. Anticonvulsant therapy including:<br>- phenytoin<br>- carbamazepine					

- barbiturates<br>- primidone<br>- topiramate<br>- oxcarbazepine<br>- lamotrigine			
17. Have you been diagnosed with tuberculosis and was on rifampicin or rifabutin therapy			
18. Major surgery with prolonged immobilisation.			
